# A new tent trap for sampling exophagic and endophagic members of the *Anopheles gambiae *complex

**DOI:** 10.1186/1475-2875-8-157

**Published:** 2009-07-14

**Authors:** Nicodemus J Govella, Prosper P Chaki, Yvonne Geissbuhler, Khadija Kannady, Fredros Okumu, J Derek Charlwood, Robert A Anderson, Gerry F Killeen

**Affiliations:** 1 Ifakara Health Institute, Coordination Office, PO Box 78373, Kiko Avenue, Mikocheni, Dar es Salaam, United Republic of Tanzania; 2 Dar es Salaam City Council, Ministry of Regional Administration and Local Government, United Republic of Tanzania; 3 Durham University, School of Biological and Biomedical Sciences, South Road, Durham, DH13LE, UK; 4 Liverpool School of Tropical Medicine, Pembroke place, Liverpool, L3QA, UK; 5 Swiss Tropical Institute, Department of Public Health and Epidemiology, PO Box 4002, Basel, Switzerland; 6 Danish Bilharziaisis Laboratory, 1-D Jaegersborg Allé, Charlottenlund, DK 2920, Denmark; 7 Biology Department, University of Winnipeg, Winnipeg, Manitoba, R3B 2E9, Canada

## Abstract

**Background:**

Mosquito sampling methods are essential for monitoring and evaluating malaria vector control interventions. In urban Dar es Salaam, human landing catch (HLC) is the only method sufficiently sensitive for monitoring malaria-transmitting *Anopheles*. HLC is labour intensive, cumbersome, hazardous, and requires such intense supervision that is difficulty to sustain on large scales.

**Methods:**

Novel tent traps were developed as alternatives to HLC. The Furvela tent, designed in Mozambique, incorporates a CDC Light trap (LT) components, while two others from Ifakara, Tanzania (designs A and B) require no electricity or moving parts. Their sensitivity for sampling malaria vectors was compared with LT and HLC over a wide range of vector abundances in rural and urban settings in Tanzania, with endophagic and exophagic populations, respectively, using randomised Latin-square and cross- over experimental designs.

**Results:**

The sensitivity of LTs was greater than HLC while the opposite was true of Ifakara tent traps (crude mean catch of *An. gambiae sensu lato *relative to HLC = 0.28, 0.65 and 1.30 for designs A, B and LT in a rural setting and 0.32 for design B in an urban setting). However, Ifakara B catches correlated far better to HLC (r^2 ^= 0.73, P < 0.001) than any other method tested (r^2 ^= 0.04, P = 0.426 and r^2 ^= 0.19, P = 0.006 for Ifakara A and LTs respectively). Only Ifakara B in a rural setting with high vector density exhibited constant sampling efficiency relative to HLC. The relative sensitivity of Ifakara B increased as vector densities decreased in the urban setting and exceeded that of HLC at the lowest densities. None of the tent traps differed from HLC in terms of the proportions of parous mosquitoes (P ≥ 0.849) or *An. gambiae s.l*. sibling species (P ≥ 0.280) they sampled but both Ifakara A and B designs failed to reduce the proportion of blood-fed mosquitoes caught (Odds ratio [95% Confidence Interval] = 1.6 [1.2, 2.1] and 1.0 [0.8, 1.2], P = 0.002 and 0.998, respectively), probably because of operator exposure while emptying the trap each morning.

**Conclusion:**

The Ifakara B trap may have potential for monitoring and evaluating a variety of endophagic and exophagic Afrotropical malaria vectors, particularly at low but epidemiologically relevant population densities. However, operator exposure to mosquito bites remains a concern so additional modifications or protective measures will be required before this design can be considered for widespread, routine use.

## Background

A myriad of mosquito sampling techniques have been developed and the sensitivity with which they sample targeted mosquito species has been evaluated under an equally diverse set of field conditions [1,2]. Effective mosquito traps are essential to monitor and evaluate malaria vector control programs [3]. Such information is vital to enable malaria control practitioners to optimize intervention strategies and tactics under practical conditions of operational programmes.

In the African context, sampling of malaria vectors relies almost exclusively upon trapping highly anthropophagic mosquitoes in and around houses, either directly before or soon after feeding [4]. Aside from human landing catch (HLC), the most commonly used methods for sampling host-seeking African malaria vectors are Centers for Disease Control and Prevention miniature light traps (LTs) [5] placed beside occupied bednets. Another major strategy for trapping African malaria vectors exploits the tendency of these endophilic species to rest indoors after blood feeding [6]. Such indoor resting catches involve either aspirating directly from accessible resting places [7] or "knock down" with indoor pyrethrum spray onto white sheets where they are readily collected [8-10]. While LTs are relatively reliable [11-14] and largely unaffected by the presence of insecticidal interventions [15,16], however, methods which sample indoor-resting mosquitoes [2] are unsuitable for many control programmes because they are adversely affected by the presence of insecticides on nets or walls [17,18] which promote exit [19-21] and outdoor resting [22-24]. While exit traps placed in windows [10] have proven useful for monitoring vector density trends in southern Africa [25] and Equatorial Guinea [26], their efficiency is likely to be influenced by site and time-specific factors such as mosquito and human behaviours, as well as house design. These approaches may therefore also be unreliable for estimating representative, consistent and epidemiologically meaningful human-biting rates of vector populations.

Dar es Salaam in Tanzania is a typical, rapidly growing African city and has recently developed a large-scale programme for supplementing existing priority malaria prevention methods with systematic larviciding [27]. The microbial larvicides (*Bacillus thuringensis var israelensis*) have little residual activity, necessitating weekly application and mosquito surveillance cycles [27]. Unfortunately, none of the above mentioned trapping techniques nor a number of alternative method apart from HLC, proved sufficiently sensitive for routine mosquito surveillance. Initial attempts to use Mbita-design bednet traps [13,28,29] indoors or outdoors, yielded only one *Anopheles gambiae sensu lato *over 181 full nights of sampling. The Centers for Disease Control and Prevention miniature light traps (LTs), pyrethrum spray catch and indoor aspirator catches all failed to catch significant numbers of *Anopheles*. In stark contrast, three nights of preliminary outdoor human landing catch (HLC) at one location yielded 136 *An. gambiae s.l*. and 30 other *Anopheles*. It has since been shown through detailed behavioural studies that *Anopheles gambiae sensu stricto*) and *Anopheles arabiensis *Patton are both predominantly exophagic in this highly urbanized environment [30]. Outdoor HLC was therefore undertaken as an interim monitoring and evaluation measure while alternative outdoor trapping technologies were developed [27]. A major advantage of HLC is that mosquitoes are caught in the act of biting the human host [1,4,14,31] so the sample obtained is assumed to be representative of the human biting rate. This enables estimation of the entomologic inoculation rate (EIR) which is the average number of infective bites per person per unit time [32]. Nonetheless, this technique has major drawbacks, some of which are prohibitive. It is extremely arduous and labour intensive, requiring intense supervision to the extent that is difficult to sustain on large scales. An even greater concern arises from the fact that it inevitably increases the hazard of exposure of participants to mosquito-borne infections [2,4,10] which is difficult to justify on ethical grounds. In this article we report the development and evaluation of new tent traps in both rural and urban settings in Tanzania with very different vector population densities and behaviours.

## Methods

### Study sites

The rural study site was Lupiro village in the Kilombero Valley, 40 km south of Ifakara [16] in Ulanga district, Morogoro region, Tanzania. This valley experiences extremely high *Plasmodium falciparum *malaria transmission with an EIR exceeding 600 infectious bites per person per year despite exceptionally high coverage with largely untreated bednets [16]. The main malaria vectors are endophagic members of the *An. gambiae *complex [33].

The urban study site, Dar es Salaam is the largest city in Tanzania, situated on the Indian Ocean coast with lower transmission levels, have been proven accessible to control with larviciding and environmental management [27,34-36]. While the nocturnal biting cycle of *An. gambiae s.s*. is more or less consistent with that of classical reports [37], all members of the complex in Dar es Salaam have an unusual preference for outdoor feeding [30] and biting activity of *An. arabiensis *peaks at about 10 pm when many residents are often still awake and outdoors [30].

In both sites, houses with open eaves were chosen in order to minimize the potential confounding effect of differences in house structure upon observations of feeding behaviour and trap efficiency. Nevertheless, we did use existing rather than standardized, purpose-built houses (often referred to as experimental huts) for these surveys so some differences between the two sites were unavoidable. In rural settings houses were constructed of mud with thatched roofs while those in Dar es Salaam, all had walls made with bricks and iron roofs.

### Trapping methods

#### Furvela tent trap

**Figure 1:**
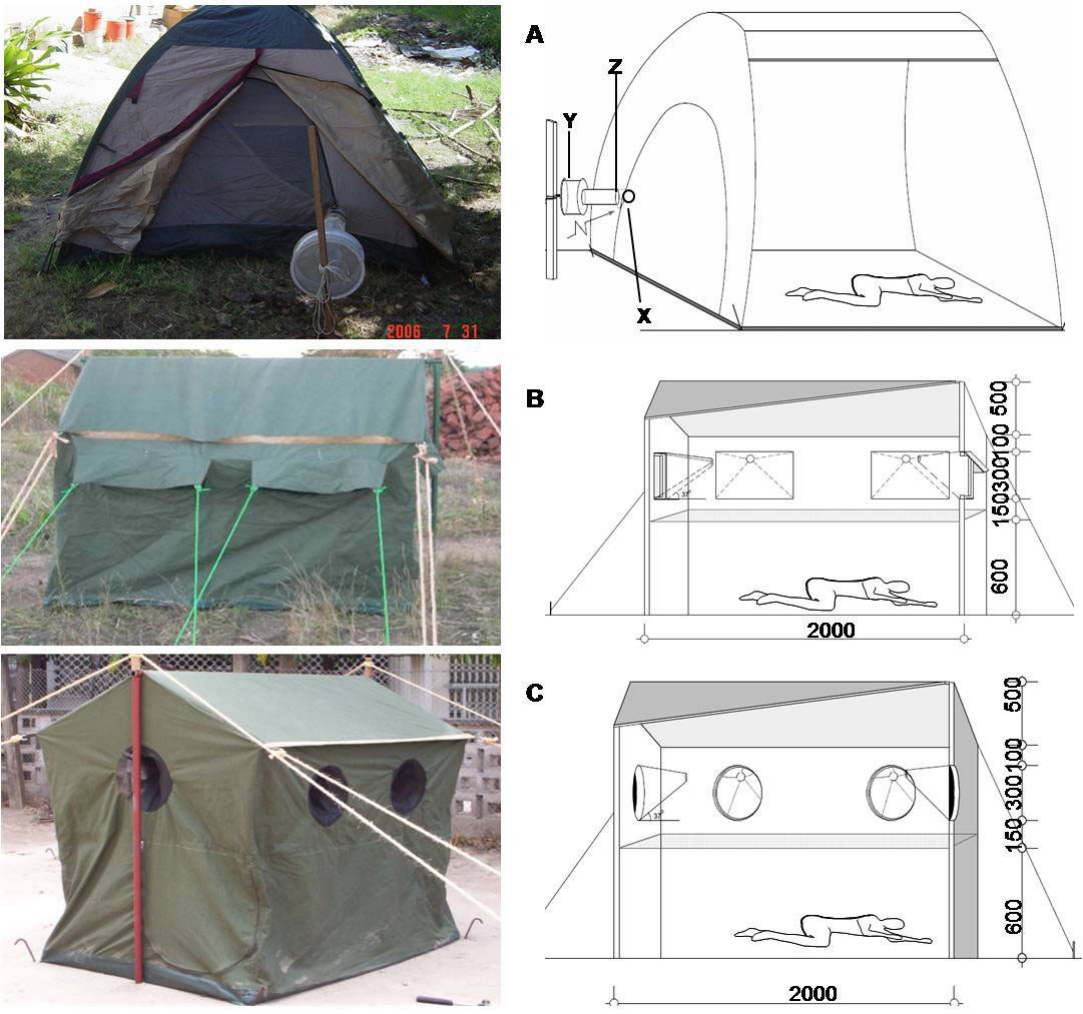
**Furvela trap (A), Ifakara A tent trap (B), Ifakara B tent trap (C), with section drawing of each**. The human occupant is protected from mosquito bites by a netting panel within the Ifakara A and B designs. For the Furvela trap, mosquitoes approach the small opening in the tent zipper (X) where they are drawn into the collection bag collection bag (Y) when they pass the CDC light trap entrance (Z), while in the Ifakara designs they enter through a funnel shaped entrances tilted upward. All dimensions in mm.

The Furvela tent trap (Figure 1A) developed and tested by one of the authors (JDC) in Mozambique, is constructed from a dome shaped Eureka^® ^sleeping tent with nylon taffeta body and floor surface. A standard LT with the light bulb removed is attached to the zip of the main tent door which is almost closed, leaving a 5 cm gap (Figure 1A: X) for host odours to escape and mosquitoes to attempt entry. This trap is powered by a 6 V battery kept inside the tent and mosquitoes are caught into the collection bag (Figure 1A: Y) through suction created by a rotating fan that is positioned near the tent entrance (Figure 1A: Z), which is in turn oriented away from the wind.

#### Ifakara tent trap

The Ifakara A and B tent traps (Figure 1B and 1C) are rectangular canvas boxes containing six funnel-like entrances for mosquitoes and inner small apertures tilted to an angle so that mosquitoes have to fly upward to enter the trap. Such baffled entrance structures are known to increase the probability that mosquitoes do not exit once inside traps [38] and this was also found to be the case in this specific example during development. A layer of durable, Teflon-coated woven fibreglass netting between the entry funnels and the bait host allows the human participant to sleep while protected from mosquito bites. Bisecting the protective netting panel, a zip enables the participant to aspirate mosquitoes from inside the trap. The trap floor is made of thick polyvinylchloride sheeting, which protects against rough substrates and surface water. The two traps differ only in the design of the entry points. Ifakara A used square shaped entrances that were partially covered by an over-hanging flap of canvas, while Ifakara B used completely exposed circular entrances (Figure 1B and 1C). These two designs, based on a prototype used previously to assess mosquito behaviour in the Kilombero valley [39], were developed iteratively in Lupiro village where very high densities of *An. gambiae s.l*. allowed rapid assessment through a series of stepwise modifications.

#### Centers for Disease Control and Prevention miniature light traps (LTs)

CDC miniature light traps (model 512) with inflorescent bulbs were each hung inside a house near an occupied, insecticide-free bednet with the top of the shield pan approximately 150 cm from the floor surface, placed at the end where the occupants feet lie and touching one side of the net [40].

#### Human landing catch (HLC)

To conduct (HLC), each adult male collector exposed his lower limbs and collected the mosquitoes when landing on his legs with an aspirator [2]. HLC was conducted by a single catcher at each station (site or house × indoor or outdoor position) for 45 minutes each hour, allowing 15 minutes break for rest. To obtain full hourly biting densities, the catches for each hour were therefore divided by 0.75 [30]. Collections were conducted both indoors and outdoors in accordance with the relevant experimental designs described below.

### Experimental design

#### Experiment 1 (rural)

**Figure 2:**
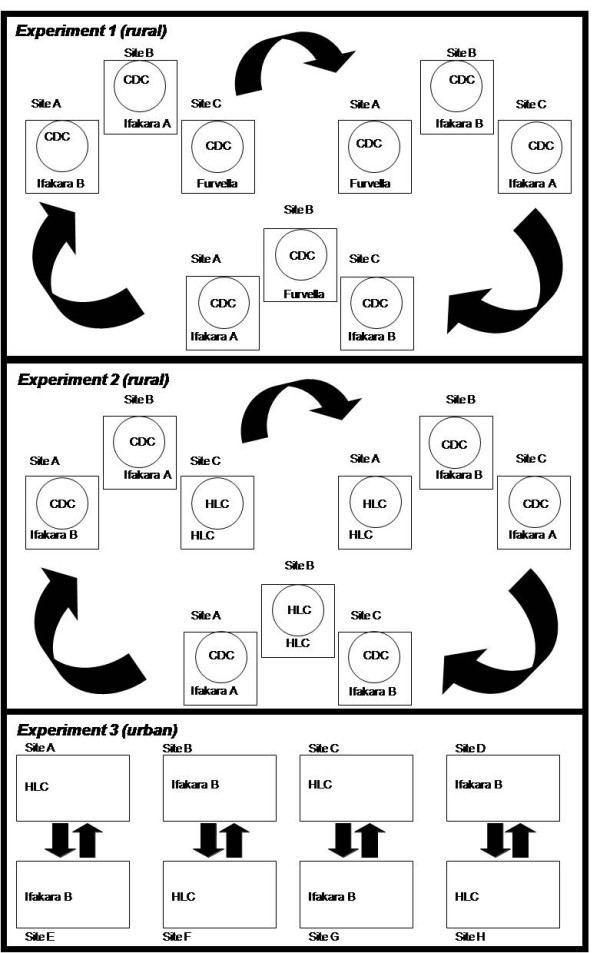
**Schematic representation of a typical experimental design indicating three possible arrangements for one complete rotation in experiment one and two with cross over design in experiment three**. Indoor and outdoor catching stations/sites are presented by circles and squares respectively.

Three houses with three corresponding outdoor catching stations immediately beside them, approximately 5 m away from a house, were selected. The Furvela, Ifakara A, and B tent traps were assigned to one of the three outdoor catching stations and rotated in order through 6 rounds of a 3 × 3 Latin square experiment design (Figure 2) so that we could directly compare each trap design with the LTs placed inside of all three houses as the reference method. The human subject assigned to each station remained fixed throughout the experiment in order to minimize the bias of differential individual attractiveness and particular locations and to combine these heterogeneities into single quantifiable source of variation. This experiment was carried out over 18 nights (8^th ^November to 25^th ^November 2006), during the short rains, constituting six full rotations of each of the three trap-site combination. Mosquitoes were collected by all methods from 19.30 to 05.30 h.

#### Experiment 2 (rural)

Experiment 2 was adapted from experiment 1 with slight changes. At one house, the pairing of the LT indoors with the Furvela tent trap outdoor was replaced by HLC, both indoors and outdoors (Figure 2) so that the Ifakara A and B tent traps could be compared with two reference methods. This experiment again relied upon a Latin square design and was implemented during the short rains (27^th ^November to14^th ^December 2006) in similar fashion to experiment 1.

#### Experiment 3 (urban)

In urban Dar es Salaam, Ifakara B traps were compared directly with HLC only. Four well-separated sites (> 100 m apart), each consisting of a pair of outdoor catching stations approximately 50 m apart, were selected with each being associated with a nearby house approximately 5 m away. Each catcher was allocated to and remained associated with a specific sampling station. On each experimental night, one participant at one of the two stations in each of the four sites conducted HLC while the other within the same site slept in an Ifakara B trap. The trapping techniques were swapped between the two stations within each site every night (Figure 2) from 23^rd ^April to 21^st ^June 2007 during the main rainy season and mosquitoes were collected by both methods from 19.30 to 05.30 h. This experiment proceeded for 41 nights with exception of one night during which data was discarded due to ants destroying some of the samples.

### Processing of samples

Mosquitoes from all catches were sorted, counted and their abdominal status (unfed, part fed, fully fed, and gravid) classified directly in the field. The abdominal status was determined in order to test whether the alternative trapping methods, tent traps in particular, reduce the exposure of participant to mosquito bites. *Anopheles gambiae s.l., An. funestus*, and other Anophelines were identified morphologically [37,41] with the aid of a stereo-microscope and as many freshly caught specimens of *An. gambiae s.l*. as possible were dissected to determine parity [42]. All mosquito samples were stored in tubes with desiccated silica for subsequent polymerase chain reaction (PCR) assay [43] to determine the sibling species of *An. gambiae *complex. Although these mosquitoes were also retained for sporozoite infection status determination, these samples were accidentally discarded following freezer failure before laboratory analysis could be completed. All Culicines were counted, categorized as male or female and discarded.

### Data analysis

#### Density-independent sampling efficiency

It is vital to measure whether the novel alternative sampling methods collects the same fraction of mosquito population as the reference method. However, the precise comparison between two sampling methods is generally difficult because errors exist in both methods and neither can be assumed to constitute a truly independent variable [44]. Bearing this in mind, we decided to undertake a diverse series of analyses to check the consistency of outcomes based on the size of female *An. gambiae s.l*. catches. Low catches of *An. funestus *were obtained in all experiments so, although it is an important vector of malaria in Tanzania and elsewhere in Africa, we cannot report a rigorous evaluation of how well these traps sample this vector. All analyses were conducted using SPSS 15.0

We first aggregated catches of female *An. gambiae s.l*. by trap type and date in experiment 3 where multiple traps of the same type operated simultaneously, yielding consistently non-zero mean catches for each trap on each night. This is an important step as it eliminates the possibility of biasing analyses of sampling sensitivity with logarithmically-transformed data which would otherwise have to be artificially converted to non-zero values by adding one [45].

Initially, simple Pearson correlation was applied using logarithmically transformed data (log_10 _(*x*)) of female *An. gambiae s.l*. from each trap. This was then complemented by plots of the catches in alternative traps against the reference group using absolute catches. Subsequently, the dependence of the sampling efficiency of the alternative tent traps, relative to the LT or HLC reference method, upon vector density and experiment was evaluated by fitting the following model using generalised estimating equations (GEE).



Where *y *is the relative sampling efficiency of the alternative technique on each night, estimated by dividing the alternative trap catch by that of the reference method, *x*_1 _is the logarithm of the catch with the reference technique, *x*_2 _is a categorical variable reflecting the identity of the experiment and *β*_*o *_is the estimated intercept reflecting sampling efficiency at an infinitesimally low vector density as measured by reference method, while *β*_1 _and *β*_2_, are the estimated parameters reflecting the influence of *x*_1 _and *x*_2 _respectively. The catch of the alternative collection methods divided by the catch of the reference method on each experimental night was therefore treated as the dependent variable with a gamma distribution in all fitted models. Site and, where appropriate, station were treated as subject effects with experimental night distinguishing repeated measures. Initially, experiment and the log-transformed catch in the reference trap were included as factor and covariate variables, respectively, in a model fitted to the pooled data from all experiments relevant to that alternative-reference method pairing. The influence of experiment was found to be significant in all cases so data from each experiment were then analyzed separately and the experiment term was removed from the model. If the influence of the log-transformed reference trap catch in such an experiment-specific initial model was not significant, indicating constant sampling efficiency across the range of vector densities within that experiment, this term was removed and the simplest model possible, with only an intercept, was fitted. The best-fit models for each experiment were then plotted and compared with the actual nightly catch data, plotted as recorded catches of the alternative collection methods divided by the recorded catch of reference methods against the catch of reference method using absolute catch numbers.

#### Distribution of parity, species and abdominal conditions among sampling techniques

The influence of collection method upon the distribution of parity, sibling species and abdominal condition of *An. gambiae s.l*. were analyzed by logistic regression, treating each as a binary outcome variable with experiment and trap design as independent categorical factors in the model. The results of dissections, PCR species determination and visual inspections upon collection were expressed in a binary fashion as being parous versus nulliparous, *An. gambiae s.s*. versus *An. arabiensis *and partly or fully blood fed versus unfed, respectively.

### Ethical clearance and protection of human participants

Prior to any field work, research clearance was obtained from the institutional review board of Durham University in the UK, Ifakara Health Institute in Tanzania, and the Medical Research Coordination Committee of the National institute of Medical Research in Tanzania (Reference numbers NIMR/HQ/R.8a/Vol.IX/279 and 324). The written informed consents were obtained from all participants. These volunteers were screened weekly for malaria parasites and, when positive, offered the best medication available, namely artemisinin-lumefantrane, (Co-Artem^®^), free of charge.

## Results

### Crude relative sensitivity of tent traps and correlation withreference methods

**Figure 3:**
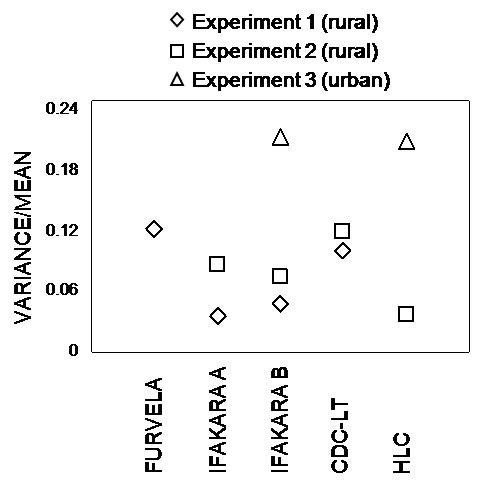
**Illustration of the relative precision for different methods in sampling *An. gambiae s.l*. across different experiments**.

**Figure 4:**
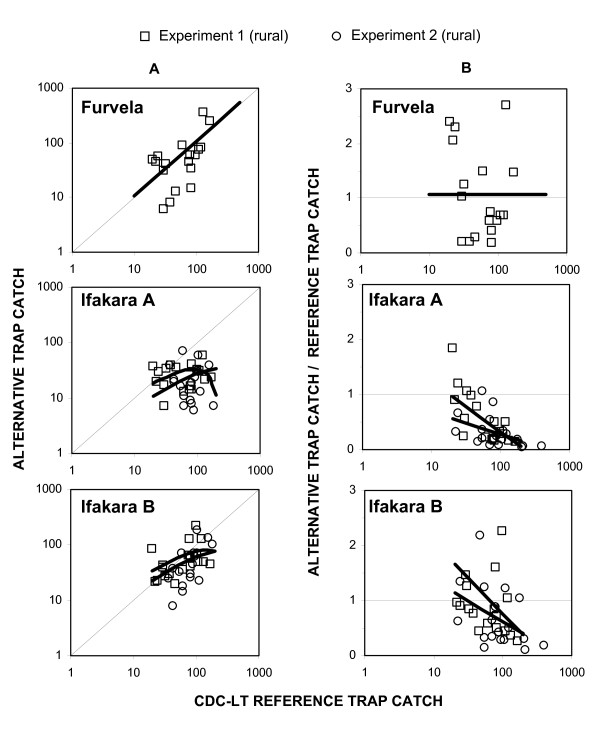
**Correlation and density-dependence of alternative methods sampling efficiency, relative to the light trap reference method for catching *An gambiae s.l.***. The correlation between the catches of *An. gambiae s.l*. in alternative methods and the light trap reference method is plotted using absolute catches is presented in the left hand panels with a thick line representing the best model fit. Right panels illustrate density-dependence by plotting the alternative method catches divided by corresponding catches in light traps against the absolute catches in the light trap.

**Figure 5:**
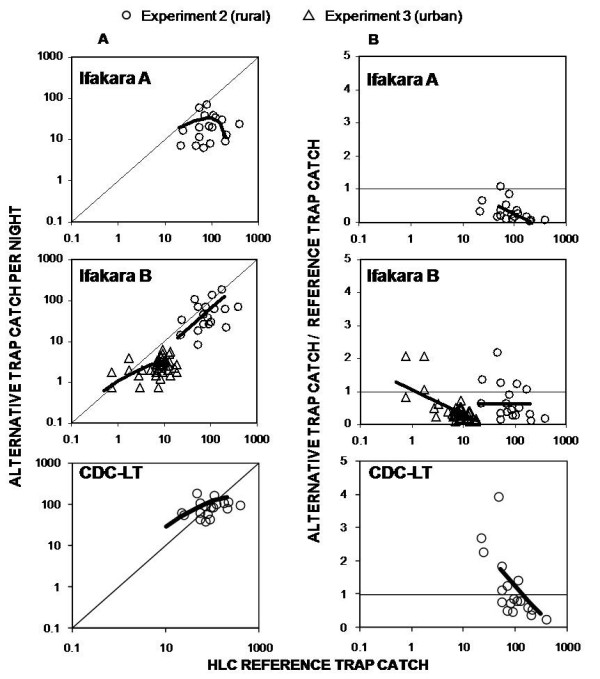
**Correlation and density-dependence of alternative methods sampling efficiency, relative to human landing catch (HLC) gold standard reference method for catching *An. gambiae s.l.***. The correlation between the catches by alternative methods and HLC is presented in the left hand panels. Right panels illustrate density-dependence by plotting catches with alternative methods divided by corresponding catches by HLC against the absolute catches in HLC.

The number of *Anopheles *trapped by each sampling method in each experiment is shown in Table 1. Based on the mean catch sizes described in Table 1, the Ifakara tent traps consistently caught less mosquitoes than the reference LTs and HLC methods. Nevertheless, even these lower mosquito catches are encouraging, because these designs do not require electricity. Comparing the quotient of variance divided by mean, shows that LTs and Furvela traps appeared to be less precise than HLC or Ifakara tent traps in the rural setting for sampling *An. gambiae s.l*. (Figure 3). The catches of the Ifakara B but not the Ifakara A trap were loosely correlated with those of the LTs (Table 2 and Figure 4). However catches by the Ifakara B design were correlated closely to those of the HLC gold standard. In fact this correlation was far stronger than that of the LTs in this study and at least matches any other previously reported evaluation of the LTs (Table 2 and Figure 5). In fact, examination of Figure 5 prompted us to restrict this linear correlation of data from pooled experiments to HLC catches of 10 per person per night or more because the relationship appears to be linear across both experiments within this range. This analysis restricted to reasonably high vector densities yielded even more encouraging results (r^2 ^= 0.86, P < 0.001).

**Table 1:** Number of *Anopheles *mosquitoes caught by different techniques relative to human landing catch

**Collection methods**		**Trap nights**	**Total catch**	**Mean catch**	**Relative sensitivity**
***Anopheles gambiae *s.l.**					
*Furvela*					
	Experiment 1	18	1306	72.6	NA
*Ifakara A*					
	Experiment 1	18	483	26.8	NA
	Experiment 2	18	429	23.8	0.28
*Ifakara B*					
	Experiment 1	18	1099	61.1	NA
	Experiment 2	18	1007	55.9	0.65
	Experiment 3	164	442	2.7	0.32
*Light trap*					
	Experiment 1	54	3736	69.2	NA
	Experiment 2	36	4008	111.3	1.30
*HLC*					
	Experiment 2	36	3081	85.6	NA
	Experiment 3	164	1398	8.5	NA
***Anopheles funestus***					
*Furvela*					
	Experiment 1	18	2	0.11	NA
*Ifakara A*					
	Experiment 1	18	2	0.11	NA
	Experiment 2	18	2	0.11	0.28
*Ifakara B*					
	Experiment 1	18	3	0.16	NA
	Experiment 2	18	4	0.22	0.55
	Experiment 3	164	13	0.07	1.40
*Light trap*					
	Experiment 1	54	21	0.38	NA
	Experiment 2	36	24	0.68	1.70
*HLC*					
	Experiment 2	36	14	0.40	NA
	Experiment 3	164	8	0.05	NA

NA: Not applicable

**Table 2:** Correlation of numbers of female *Anopheles gambiae *complex caught by alternative traps with reference collection methods, pooling data from all experiments in which simultaneously data for each pair was collected

	Alternative collection methods	versus CDC-light trap reference method		versus human landing catch reference method	
			
This study		r^2^	P	r^2^	P
					
	Furvela	**0.303**	**0.021**	NA	NA
	Ifakara A	0.008	0.590	0.040	0.426
	Ifakara B	**0.148**	**0.020**	**0.731**	**< 0.001**
	Light trap	NA	NA	**0.192**	**0.006**
Ref [14]	Light trap	NA	NA	0.723	< 0.001
Ref [46]	Light trap	NA	NA	0.409	< 0.001
Ref [48]	Light trap	NA	NA	0.476	< 0.001
Ref [15]	Light trap	NA	NA	0.521	< 0.001

NA: Not applicable

### Density-dependence of trap sampling efficiency

Compared to the LT reference method, the Furvela trap was the only alternative method which showed density-independent sensitivity (Table 3 and Figure 4). Consistent with Figure 3, this method yielded catches which were imprecise but otherwise almost exactly equivalent to those of the LTs, suggesting their common components and mechanisms of action may result in similar sampling characteristics and dependence upon confounding factors. In contrast, both Ifakara A and B were clearly less sensitive at high vector densities when compared to LTs (Table 3 and Figure 4), but at low densities the Ifakara B design was at least as sensitive as the LT. Similarly, only one instance of constant sampling efficiency was apparent when HLC was treated as the reference group. All alternative traps, with the exception of Ifakara B in experiment 2, proved to be more sensitive at low vector densities and decrease with increasing vector abundance (Table 3 and Figure 5). Although the sensitivity of the Ifakara B trap increased with decreasing vector density in experiment 3, it is noteworthy that, again this alternative exceeds the sensitivity of the reference method at the lowest vector densities. Given that the HLC is considered a more reliable gold standard than LT, these observations again strengthen the case that the Ifakara B trap is probably the most reliable, if not always the most sensitive, of the alternative traps evaluated here as surrogates of human exposure to malaria vectors.

**Table 3:** Density-dependence of relative sampling efficiency of alternative traps for *An. gambiae s.l*. by generalized estimating equations (GEE)

Alternative collection method		Parameter	Estimate [95%CI]	P
			
***Versus CDC-light trap reference method***				
*Furvela*				
	Experiment 1	Intercept	1.07 [0.70, 1.44]	< 0.001
*Ifakara A*				
	Experiment 1	Intercept	2.12 [1.42, 2.82]	< 0.001
		Log_10_(CDC-LT)	-0.90 [-1.22, -0.57]	< 0.001
	Experiment 2	Intercept	1.05 [0.59, 1.51]	< 0.001
		Log_10_(CDC-LT)	-0.39 [-0.57, -0.21]	< 0.001
*Ifakara B*				
	Experiment 1	Intercept	3.31 [1.17, 5.45]	0.002
		Log_10_(CDC-LT)	-1.27 [-2.35, -0.19]	0.021
	Experiment 2	Intercept	2.10 [1.22, 2.99]	< 0.001
		Log_10_(CDC-LT)	-0.74 [-1.09, -0.39]	< 0.001
	Experiment 3	NA	NA	NA
*Light trap*				
	Experiment 2	NA	NA	NA
***Versus human landing catch reference method***				
*Furvela*				
	Experiment 1	NA	NA	NA
*Ifakara A*				
	Experiment 1	NA	NA	NA
		NA	NA	NA
	Experiment 2	Intercept	1.69 [0.86, 2.52]	< 0.001
		Log_10_(HLC)	-0.71 [-1.09, 0.33]	< 0.001
*Ifakara B*				
	Experiment 1	NA	NA	NA
		NA	NA	NA
	Experiment 2	intercept	0.64 [0.46, 0.81]	< 0.001
	Experiment 3	Intercept	1.06 [0.78, 1.33]	< 0.001
		Log_10_(HLC)	-0.75 [-0.99, 0.50]	< 0.001
*Light trap*				
	Experiment 2	Intercept	4.65 [1.58, 7.71]	0.003
		Log_10_(HLC)	-1.71 [-3.243, -0.178]	0.029

### Influence of trap design on the parity, species and abdominal status distribution

Table 4 compares the parity status distribution of *An. gambiae s.l*. sampled with the various alternative methods with that of the HLC gold standard. No significant differences were noted for any of the trapping methods. Although the raw data might suggest different parity rates in samples obtained with the various trapping methods, this arises from their differential distribution across experiments 1 and 2 which sampled populations with very different age structures (Table 4). The lack of differences between alternative methods and HLC suggests they all represent reasonable options for sampling mosquitoes to determine the age distribution, and therefore the infection status, of the host-seeking vector population. Furthermore, the sibling species composition of the *An. gambiae s.l*. revealed that *An. gambiae s.s*. and *An. arabiensis *were the only subspecies obtained from successfully (n = 3136) amplified specimens and LT was the only method which differed from HLC (Table 5). The LT oversampled *An. gambiae s.s*.

**Table 4:** The influence of trapping method and experiment upon the proportion of sampled *An. gambiae s.l*. which were parous, determined by logistic regression as described in the methods section

Variable	Parous (%)	OR [95%C.I]	P
			
*Trap type*			
Furvela	22.2 (35/158)	0.89 [0.55, 1.45]	0.849
Ifakara A	30.5 (68/223)	0.99 [0.55, 1.45]	0.957
Ifakara B	30.4 (106/349)	1.00 [0.73, 1.36]	0.999
Light trap	15.2 (141/930)	0.97 [0.74, 1.27]	0.849
Human landing catch	41.0 (293/714)	1.00^a^	NA
			
*Experiment*			
Experiment 1	23.6 (168/713)	0.51 [0.39, 0.67]	< 0.001
Experiment 2	28.6 (475/1661)	1.00^a^	NA

**Table 5:** The influence of trapping method and experiment upon the proportion of sampled *An. gambiae s.l*. which were *An. gambiae s.s*., determimed by logistic regression as described in the methods section

Variable	*An. gambiae s.s*(%)	OR [95%C.I]	P
			
*Trap type*			
Ifakara A	8.2 (12/146)	0.71 [0.39 1.32]	0.28
Ifakara B	61.3 (234/382)	0.84 [0.49 1.41]	0.50
Light trap	14.3 (116/814)	1.32 [1.02 1.71]	0.03
Human landing catch catch	32.9 (591/1794)	1.00^a^	NA
			
*Experiment*			
Experiment 2	11.9 (294/2471)	0.001 [0.001 0.002]	< 0.001
Experiment 3	99.1 (666/672)	1.00^a^	NA

NA: not applicable

Over 89% of *An. gambiae s.l*. caught with each method over all experiments were unfed. This suggests that each method used in these experiments predominantly sampled host-seeking vectors. The proportions of mosquitoes caught with each method and in each experiment which were fully or partly blood fed are presented in Table 6. Both the Furvela and LT which rely on similar components and mechanisms, catch far fewer blood-fed mosquitoes than HLC. This confirms that these are indeed exposure-free methods which prevent the frequent occurrence of blood feeding upon the catcher before capture, as is inevitable when conducting HLC. The lack of consistent differences between the Ifakara designs and HLC suggests that exposure does occur when sampling with this trap, most probably when the zip is opened in the morning and the operator aspirates from inside the trap chamber.

**Table 6:** The influence of trapping method and experiment upon the proportion of sampled *An. gambiae s.l*. which were fully or part blood fed, determined by logistic regression as described in the methods section

Variable	Proportion fed (%)	OR [95%C.I]	P
			
*Trap type*			
Furvela	1.53 (20/1306)	0.24 [0.15, 0.39]	< 0.001
Ifakara A	10.90(47/429)	1.56 [1.17, 2.10]	< 0.002
Ifakara B	6.51 (166/2548)	1.00 [0.80, 1.23]	0.998
Light trap	1.83 (142/7744)	0.32 [0.25, 0.40]	< 0.001
Human landing catch	8.10 (363/4479)	1.00^a^	NA
			
*Experiment*			
Experiment 1	3.10 (204/6624)	0.56 [0.43, 0.72]	< 0.001
Experiment 2	3.74 (319/8525)	0.48 [0.39, 0.58]	< 0.001
Experiment 3		1.00^a^	NA

## Discussion

The use of mosquito trapping techniques to estimate daily vector biting rates experienced by humans requires not only that such approaches are sufficiently sensitive, but also that sampling efficiency is known. The relative sampling efficiency of LT was found to be density-dependent with its efficiency decreasing at high vector densities. This finding supports other reports from areas of low malaria vector density in Kilifi on the coast of Kenya for *An. gambiae s.l*. [46] and in Papua New Guinea for *An. punctulatus *and *An. farauti *[47]. In other studies, however, the relative sampling efficiency of LT has been found to be density-independent [14,15,48,49]. Unlike the Kilifi study [46], no zero values were present in the aggregated data and no transformation other than logarithm were necessary. Therefore, this density-dependence cannot be attributed to mathematical artifact [45] and appears to be a genuine property of the sampling device. Note that our estimate of mean relative sensitivity for the LT differs from previous trials in the same Tanzanian village [50] and re-analysis of this data reveals the same density-dependence for LT that we have outlined here (Okumu unpublished). The apparently variable trapping efficiency of LT between and within studies, may not necessarily be due to differences in statistical approach [15] but rather to subtle and intensely heterogeneous factors which inevitably vary through space, time and investigation. Such essentially uncontrollable factors could include the positioning of the paired techniques, use of interventions such as bednets and insecticides, lunar phase, season, weather and house architecture. For instance, one study in Papua New Guinea [47] reported that the relative sampling efficiency of LT placed indoor was independent of outdoor *An. bancroftii *density changes and simultaneously density-dependent in relation to indoor vector abundance while the reverse trend was true for *An. longirostris*. One study in Africa [15] noted some evidence that the presence of treated nets reduce the relative sampling efficiency of LT but this effect was slight and in other similar studies [16,51] no effect could be demonstrated. Several previous studies concluded the LT to be free of age-related sampling biases [14,48,52] consistent with our observations but not those of other reports [53,54]. The difference between these studies might be partly explained by variability in both the position of LT relative to the floor [40], the quality of net [14], and variability in sleeping behavior of net occupants [55].

Here the sampling efficiency of Ifakara B traps relative to HLC has been evaluated in two very different eco-epidemiological settings, where malaria transmission intensity ranges from less than one [34] to over 600 [16] infectious bites per person per year and the vector species in question have clearly distinct feeding behaviors and activity patterns [30,33]. The sampling efficiency of the Ifakara B design appeared to be independent of vector density in the rural area with high vector abundance but appeared to increase at low densities in the urban setting, possibly reflecting reduced attentiveness of HLC catchers at low mosquito densities. Clearly none of these entomological techniques are precise, accurate or representative of true human biting rates but it is encouraging that the Ifakara B design correlates quite well to the HLC, given its lack of dependence on electricity, access to the inside of houses or intensive effort, plus its increased sampling efficiency at low densities. Also, the modest sampling efficiencies indicated by this crude analysis are sufficiently high to suggest these tent trap designs could be useful for extensive, sustained vector surveillance because of their lower cost and difficulty per trap night of sampling.

The observation that the proportion of fed mosquitoes caught in the tent traps is at least as high as for those caught by HLC implies that either these traps act as resting shelters for freshly fed mosquitoes or that the human bait actually does get bitten while aspirating mosquitoes. We have occasionally observed the latter process occurring in practice and suggest a relatively clear avenue for improvement to develop a design which truly is exposure-free and completely protects the user from exposure to mosquito bites. We conclude that the Ifakara B tent trap may be a valuable tool for large-scale surveillance, particularly in resource-limited settings if concerns about operator exposure while collecting each morning could be overcome through modification or protective measures.

The new trapping method was primarily intended to replace both LTs and HLC for routine monitoring of African malaria vectors in large scale programmes such as the Dar es Salaam Urban Malaria Control Programme (UMCP). As such there is an urgent need to first adapt this tool to prevent operator exposure and then develop a protocol allowing community based volunteers to trap and submit mosquitoes to a central laboratory without the need for intensive, expensive and unsustainable level of support and supervision [30]. We nevertheless caution that all trapping methods described here, including the Ifakara B and HLC have substantial limitations as tools for measuring mosquito biting density and human exposure. Generally speaking, mosquito sampling methods remain poorly characterized and standardized [56], and as reported here, are difficult to unambiguously relate to human exposure and malaria risk. We therefore suggest that entomological measures of transmission for routine use should ultimately be evaluated in comparison with parasitological indicators of human exposure so that the most representative and epidemiologically relevant tools can be identified.

## Competing interests

The authors declare that they have no competing interests.

## Authors' contributions

NJG developed both Ifakara tent trap formats and then designed and implemented mosquito sampling protocol in collaboration with the other authors. He also performed the data collection, analysis, interpreted the results and drafted the manuscript in consultation with the other authors. PPC, YG, FOO and KK supported in design and implementation of mosquito sampling protocols. JDC designed the Furvela trap and assisted with the field evaluation protocol. RA contributed to the initial design of the Ifakara tent trap formats. GFK conceived the study and contributed to the development of Ifakara tent trap formats. He also supervised design and implementation of the mosquito sampling protocol, data analysis, interpretation of the results and drafting of the manuscript. All authors have read and approved the final manuscript.
